# Diagnosis and management of intracranial tuberculomas: about 2 cases and a review of the literature

**DOI:** 10.11604/pamj.2019.34.23.17587

**Published:** 2019-09-11

**Authors:** Farid Zahrou, Yassine Elallouchi, Houssine Ghannane, Said Ait Benali, Khalid Aniba

**Affiliations:** 1 Neurosurgery Department, Ibn-Tofail Hospital, Marrakech, Morocco; 2 Neurosurgery Department, Mohammed VI University Hospital, Marrakech

**Keywords:** Cerebral tuberculoma, treatment, magnetic resonance imaging, prognosis

## Abstract

Central nervous system tuberculosis is a major cause of morbidity and mortality in developing countries. Intracranial tuberculoma is rare and is one of the most severe cases of tuberculosis. We present two cases. The first one is about a girl of 7 years, followed for 5 months for lymph nodes tuberculosis on anti-TB treatment that presents generalized tonic-clonic seizures associated with progressive intracranial hypertension syndrome. Brain MRI has objectified necrotic nodules in left hemisphere. The surgical approach of the lesions was direct with complete excision. The diagnosis of tuberculoma was confirmed by anatomopathological examination. The second case is about a 6-year-old girl with no particular medical history, which presents for three months progressive and treatment-resistant cervico-occipital headaches associated with walking difficulties. The MRI objectified left cerebellar tumor process interpreted preoperatively as medulloblastoma. The patient was operated on intraoperative, appearance was that of a nodular lesion. Anatomopathological examination confirmed the diagnosis. The intracranial tuberculoma is an unusual variety of the central nervous system tuberculosis and remains a topical issue in Morocco. The prognosis depends on prompt diagnosis, quality of surgical resection and anti-TB treatment. The diagnostic confirmation is histological and should therefore be evoked infront of any intracranial process mimicking a brain tumor.

## Introduction

Tuberculomas represents with leptomeningitis the most common tuberculous lesions. They are responsible of 10-30% of the intracranial expansive processes in endemic countries. In tuberculous meningitis, the incidence of tuberculomas is estimated to be between 4 and 28%. It is probably underestimated since 50% of the subjects are asymptomatic.

## Patient and observation

**Figure 1:**
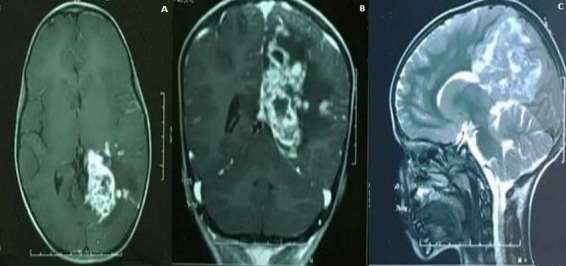
A) axial T1 weighted MR image showing left parieto-occipital necrotic nodules with high signal intensity and meningeal granulations; B) coronal T1 weighted MR image with gadolinium injection showing peripheral enhancement of the lesions; C) sagittal T2 weighted MR image showing nodules with low signal intensity and peripheral edema

**Case 1:** a seven-year-old girl, followed for 5 months for lymph nodes tuberculosis on anti-TB treatment, which was revealed by suppurating then fistulated jugular-carotid and supraclavicular lymph nodes and confirmed by ganglionic biopsy which objectified a tuberculous granuloma. Clinical evolution was marked by the appearance of generalized tonic-clonic seizures with intracranial hypertension syndrome made of treatment-resistant headaches and vomiting, all evolving in a context of fever and alteration of the general state. On admission, she was febrile (39°C), had lost 6kg in 5 months and had small cervical lymphadenopathies. Cerebral MRI objectified necrotic nodules in left hemisphere with edema and meningeal granulations (Figure 1). Biological examinations showed an accelerated sedimentation rate, microcytic hypochromic anemia, and neutrophilic leukocytosis. The intradermal reaction to the tuberculin was positive. The cyto-chemical and bacteriological analysis of the CSF objectified hypoglycorrhachic lymphocytic meningitis. The diagnosis of the cerebral tuberculoma was suggested in front of the clinical context and radiological criteria. The diagnosis of tuberculous abscess was also suspected. The surgical approach of the lesions was direct with complete excision. The histological study showed a brain parenchyma modified by a polymorphic inflammatory infiltrate with epithelioid and giant cell granuloma with caseous necrosis in favor of tuberculoma. The patient continued her anti-TB treatment. Short-course low-dose corticosteroid therapy (1mg/kg/d) was prescribed in front of perilesional edema and associated meningeal involvement. Clinical evolution was marked by the appearance of 3/5 spastic hemiparesis postoperatively recovered after 1 month of motor kinesitherapy with good clinical improvement.

**Figure 2:**
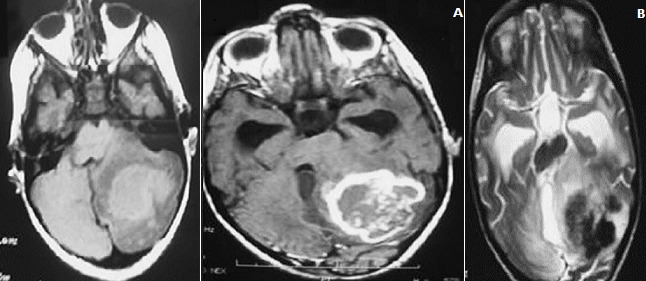
A) brain MRI in axial slices showing a process with isosignal on T1 responsible for a mass effecton vermis and V4 with upstream hydrocephalus; B) enhancing annularly after injection of gadolinium and low signal intensity on T2

**Figure 3:**
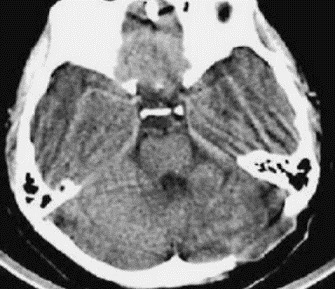
Brain CT with contrast 20 months after surgery and antibacillary treatment showing resolution of the lesion, the fourth ventricle is normally visible

**Case 2:** a six-year-old girl, with no particular medical history, hospitalized for occipitocervical headaches, resistant to analgesic treatment, associated with vomiting and walking disorders, without fever or other associated signs, all evolving in a context of weight loss of 5kg. The clinical examination found a statokinetic cerebellar syndrome. Brain MRI objectified an expansive process of the left cerebellar hemisphere with isosignal on T1, low signal intensity on T2, enhancing annularly after injection of gadolinuim and responsible for a mass effect on vermis and V4 with upstream hydrocephalus (Figure 2). The radiological image was interpreted as medulloblastoma of the posterior cerebral fossa. The patient was operated on using median suboccipital approach, the intraoperative aspect was that of a nodular and avascular lesion. The histological study showed brain parenchyma largely destroyed by granulation tissue formingepithelioid and giant cell granuloma with caseous necrosis in favor of tuberculoma. The assessment of the extent of tuberculosis was negative. The patient was put on anti-tuberculosis drugs: isoniazid (5mg/kg), rifampicin (10mg/kg), pyrazinamide (30mg/kg), streptomycin (15mg/kg) for two months then rifampicin (10mg/kg) and isoniazid (5mg/kg) for seven months. The clinical evolution was good marked by the regression of intracranial hypertension signs and the disappearance of walking disorders. A brain scan after 20 months showed the disappearance of the tuberculoma (Figure 3).

## Discussion

Tuberculosis is a major public health problem in developing countries, particularly in Morocco, where an annual incidence of 30,000 new cases/year is found, all localizations combined [1]. The manifestations of tuberculosis of central nervous system are: tuberculous meningitis which is the most frequent, followed by the tuberculoma and the cerebral abscess [2]. Tuberculomas still represent 5 to 30% of the intracranial expansive processes in developing countries. The contamination may be by haematogenous spread of systemic tuberculosis or by proximity extension of a meningeal or vertebral TB. Tuberculomas are most often supratentoriel in children, the most common site is the cerebral hemispheres. One of the patients had a subtentoriel localization [3, 4]. The symptomatology is not specific depending on the localization, the size and the number of lesions. The general signs in the weeks preceding the neurological signs are inconstant [4]. Hemispheric tuberculomas, which account for 50% of cases, are for a long time well tolerated before manifesting by convulsive seizures or a deficit syndrome (case 1), often by mass effect. They frequently sit on the parietal lobes and more often in the left hemisphere, in line with the hypothesis of anhematogenous spread of the infection more frequent in the dominant hemisphere [4]. Cerebral tuberculomas are usually associated with another tuberculous location, usually pulmonary. The pulmonary tuberulosis may be occult but responsible for the hematogenous spread of BK to the brain parenchyma [5]. Rarely, cerebral tuberculomas are associated with meningitis (case 1), which may be the source of infection or secondary to the rupture of a tuberculoma in the arachnoid space [6].

Radiological aspects of the cerebral tuberculoma are not specific. The brain scan reveals the tuberculoma as an isodense or hyperdense lesion in the calcified forms with ring enhancement after contrast injection [7]. The presence of calcifications on CT with a ring enhancement called the target sign is considered by some authors like specific of tuberculoma [8]. MRI is more sensitive than the scan for the detection of small tuberculomas and those located in the brain stem [9]. They have intermediate or low signal intensity on T1 weighted images with a ring enhancement after injection of gadolinium. On T2 weighted sequences, they usually have low signal intensity with surrounding edema [9, 10]. Basing on a series of 18 patients (ten of them were HIV positive), different aspects of tuberculomas on the MRI are described according to the evolutionary stage of the lesion. Small tuberculomas may appear like disseminated lesions with varying signal intensity, whereas mature tuberculomas are more voluminous, have a hypo-intense center and are surrounded by edema; The lesions at this stage may show nodular or ring enhancement, hence the difficulties in differential diagnosis with other tumor pathologies with the same radiological aspect like metastasis. The differential diagnosis of tuberculomas include mainly the tuberculous abscess and the tumor lesions (glioma, metastasis) when they are encapsulated and have necrotic center [10]. Differential diagnosis is different in HIV+ patients. Toxoplasmosis and lymphoma should be considered in this case [11]. The definitive diagnosis of tuberculoma is made by identifying tubercle bacillus in bacteriological samples or by a histological analysis showing a tuberculoid granuloma [12]. The direct surgical approach is justified only in case of threatening intracranial hypertension, decreased visual acuity, hydrocephalus on tuberculoma of the posterior fossa and paradoxical increase in size of tuberculoma under medical treatment [13, 14]. Postoperative mortality is about 10-20%, mostly due to postoperative meningitis [15]. In other cases, suspicion of tuberculoma is an indication of choice for stereotactic biopsy [16]. In case of small tuberculomas or tuberculous abscesses, the authors recommend medical treatment, sometimes with radiological criteria and without histological confirmation [17]. Early administration of anti-TB treatment allows to cure more than 85% of cases. Anti-TB treatment combines initially isoniazid, rifampicin, ethambutol and pyrazinamide for two months, relayed by dual therapy (isoniazid, rifampicin). In the absence of studies, the recommended duration of treatment is nine to 12 months. Corticosteroid therapy is usually used in case of extensive cerebral edema or associated meningeal involvement [4, 18].

## Conclusion

Cerebral tuberculoma is a rare but serious form of extrapulmonary tuberculosis. It's characterized by clinical polymorphism often leading to delayed diagnosis. MRI is the examination of choice for diagnosis, especially for small tuberculomas and those located in the brain stem. Its prognosis remains guarded despite the improvement of its management.

## Competing interests

The authors declare no competing interests.
